# Long-term Stability after Reduction of Mandible Fracture by Keyhole Plate: Evaluation at the Time of Plate Removal

**DOI:** 10.1186/s40902-020-00251-w

**Published:** 2020-03-16

**Authors:** Kyeong-Jun Cheon, Seoung-Won Cho, Won-Seok Jang, Ju-Won Kim, Byoung-Eun Yang

**Affiliations:** 1 Department of Oral and Maxillofacial Surgery, Hallym University College of Medicine Sacred Heart Hospital, Anyang, Republic of Korea; 2 Graduate School of Clinical Dentistry, Hallym University, Chuncheon, Republic of Korea; 3 Institute of Clinical Dentistry, Hallym University, Chuncheon, Republic of Korea

**Keywords:** Yang’s Keyhole plate, Mandibular fractures, Plate removal, Open reduction and internal fixation

## Abstract

**Background:**

Various types of miniplates have been developed and used for the reduction of facial bone fractures. We introduced Yang’s Keyhole (YK) plate, and reported on its short-term stability. The purpose of this study was to evaluate the long-term stability of the YK plate, as a follow-up study, by examining the patients who had used the YK plate among the patients with the reduction of mandible fractures and who visited for plate removal.

**Methods:**

We reviewed the medical records of 16 patients who underwent mandibular fracture fixation using a YK plate (group I) and 17 patients who underwent mandibular fracture fixation using a conventional plate (group II). Assessment was then made on malunion, occlusal stability, discomfort during the application, and clinical symptoms.

**Results:**

From January 2015 to December 2017, a total of 36 patients underwent mandibular fracture surgery using a YK plate. A total of 16 patients received plate removal. Among them, 15 were male and 1 female. The average age was 26 years. The applied surgical sites were the 12 on mandibular angle, 4 on mandibular symphysis, and 2 on subcondyle. The application period of YK plate was an average of 335 days. During the same period, 45 people underwent surgery on the conventional plate. A total of 17 patients received plate removal. Among them, 15 were male and 2 females. The average age was 36 years. The applied surgical sites were the 8 on mandibular angle, 4 on mandibular symphysis, and 2 on subcondyle. The application period of the conventional plate was an average of 349 days. No malocclusion occurred at the time of removal, and occlusion was stable. No patient complained of joint disease or discomfort.

**Conclusion:**

The YK plate system, in which the screw was first inserted and the plate was applied, for clinical convenience did not cause any particular problem and no significant difference from the conventional plate.

## Background

Currently, the plate system is the most popular treatment method for mandibular fractures using open reduction [1–3]. The plate system was first developed by Hansmann in 1886 as a treatment for iliac fractures in orthopedic surgery [4, 5]. Luhr then published the principle of open reduction and internal fixation for mandibular fracture in 1968 [6], and the first plate was made of Vitallium. It was difficult to adjust and was gradually replaced by stainless steel. In 1969, the Association for Osteosynthesis/Association for the Study of Internal Fixation (AO/ASIF) used titanium plate for the first time in limb surgery [7]. After Luhr, Michelet first applied a miniplate using a monocortical screw [8]. In 1979, Champy further developed Michelet’s method, claiming the position of an ideal plate considering the tension in the mandibular fracture, and establishing the basis of the biomechanical treatment of the plate system [9].

The development of miniplate was mainly on the improvement of the material and the location and method of application, and the development of the shape of miniplate was very little. The form of the miniplate is mostly a circular hole with a screw through the straight body. Depending on the location, there are different lengths, L-shaped, X-shaped, etc. However, the design of the circular holes through which the screw passes is all similar. In case of a miniplate of such a circular hole design, the miniplate is located at the fracture site first, and then the screw is applied to the operation. It is not easy to apply the plate in the mandibular angle area or the subcondylar area because of the reduced visual field [10, 11]. Thus, we have transformed the hole shape of the miniplate into a keyhole shape to solve this inconvenience.

This plate was named Yang’s Keyhole (YK) plate and its short-term stability was reported [12]. Surgery using this plate is well described in previous reports [12]. In the area where this plate will be applied, the first screw is inserted about 2/3 of the total length. The keyhole of the plate then allows the head of the screw to pass through and the plate is pulled into the fracture line. After the screw is inserted into the remaining holes of the plate, the entire length of the first screw inserted is completely inserted. If further fixing is required, a screw with a wide head is inserted in the remaining part of the keyhole area. Therefore, if the YK plate is used for surgery, the screw is applied first. In the case of the screw first operation method, it is allowed to fix the position of the plate quickly by using the screw applied to the first position, and it is possible to reduce the operation time by increasing the convenience of the operator. We have already reported on short-term stability [12]. The purpose of this study was to evaluate the long-term stability of the YK plate by comparing the patients who underwent plate removal patients using a conventional plate and YK plate.

## Material and methods

**Fig. 1:**
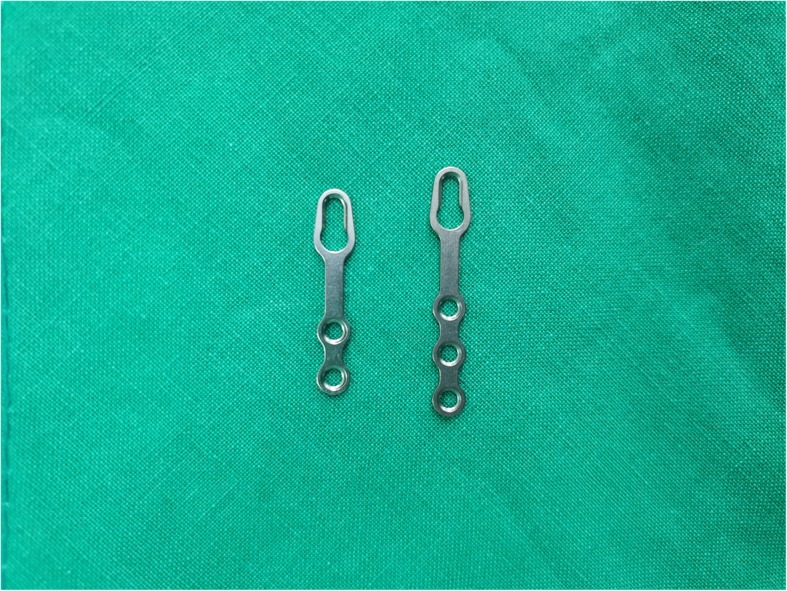
YK plate

**Fig. 2:**
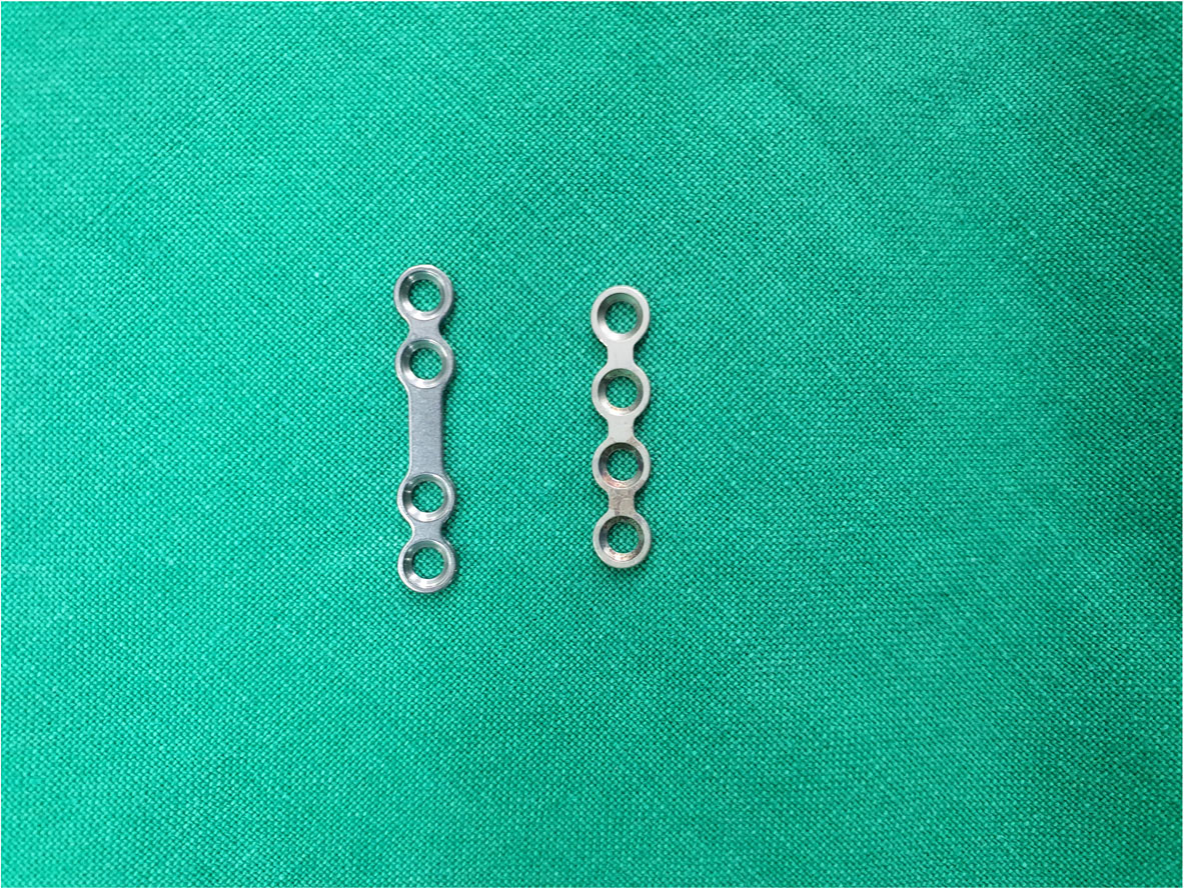
Conventional plate

**Fig. 3:**
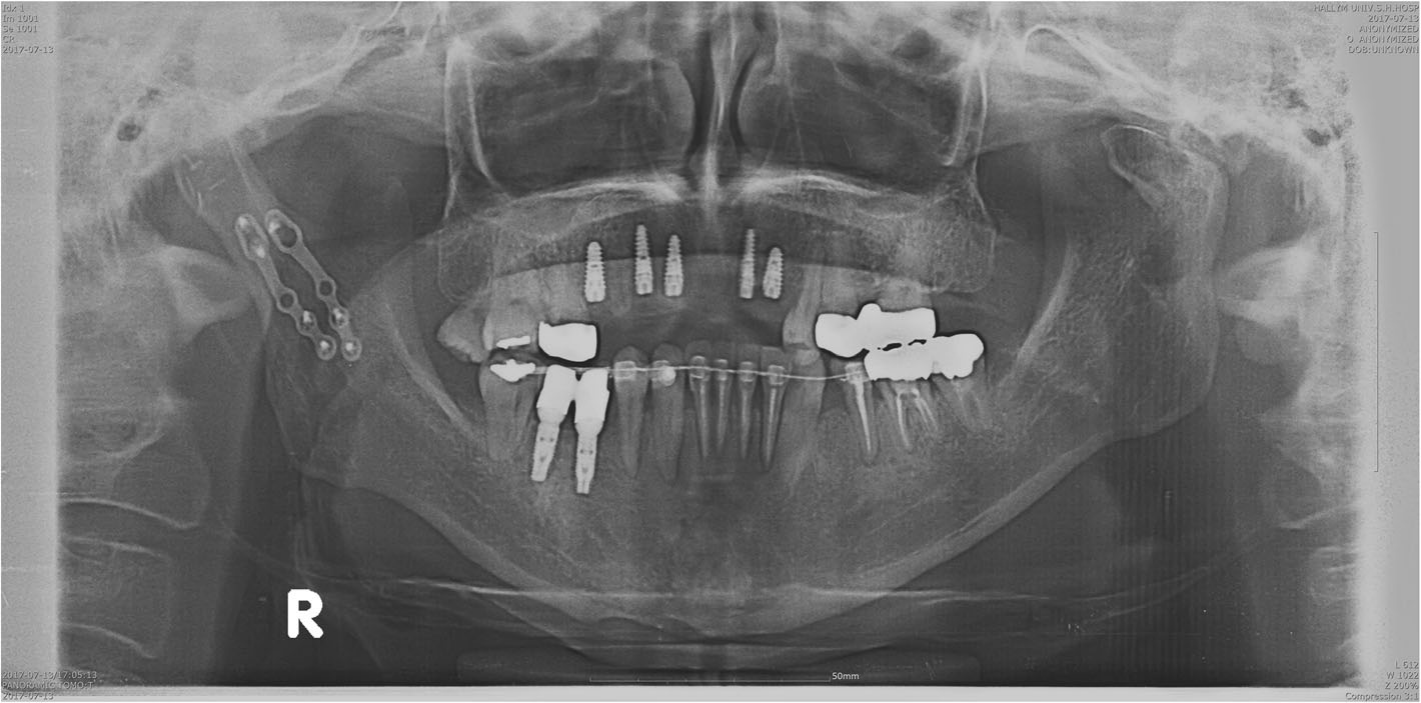
Postoperative panoramic radiographs treated with YK plate

**Fig. 4:**
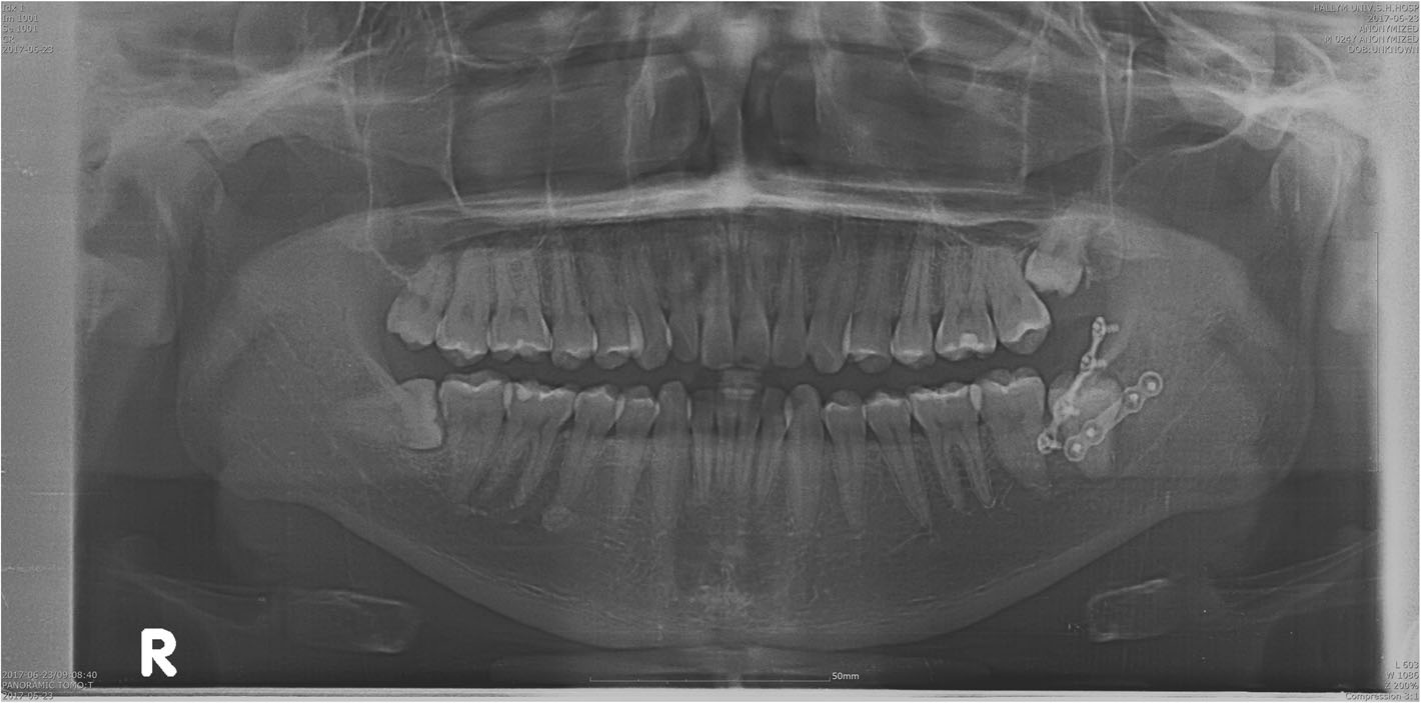
Postoperative panoramic radiographs treated with conventional plate

We reviewed the medical records of 33 patients who underwent plate removal from January 2015 to December 2017 at the Department of Oral and Maxillofacial Surgery, University Hospital. All of these patients have undergone an open reduction of mandibular fractures with YK plate (Osteonic Co., Seoul, South Korea) (Fig. 1) and conventional plate (Osteonic Co., Seoul, South Korea) (Fig. 2) in the same hospital in the past. Patients were divided into two groups. Group I is patients who underwent open reduction of the mandibular fracture using YK plate and plate removal (Fig. 3). Group II is patients who underwent open reduction of the mandibular fracture using a conventional plate and plate removal (Fig. 4).

 Comparison between two groups was made based on seven categories of complications: (1) whether the plate is broken, (2) whether the screw is loosened, (3) TMJ discomfort, (4) malunion, (5) infection, (6) abnormal occlusion, and (7) sensory abnormality. Patients were assessed before, after, and 1 week after the plate removal surgery. Radiographic evaluation was performed by photographing the panoramic radiograph immediately after the operation. Retrospective clinical study was approved by Hallym University Sacred Heart Hospital’s institutional review board (IRB No. 2018-05-013-001). All clinical study was performed in accordance with the relevant guidelines and regulations—retrospective unnamed data collected for the evaluation purpose.

## Results

From January 2015 to December 2017, a total of 81 patients underwent open reduction due to mandibular fracture in an oral maxillofacial surgeon of Sacred Heart Hospital, Hallym University. Of them, 36 patients were using the YK plate and 45 patients were using a conventional plate. Sixteen patients (group I) underwent plate removal of the plate using a YK plate. Among the 45 patients who were used conventional plate, 17 patients (group II) received plate removal. Of group I patients, 15 were male and 1 was female. Of group II patients, 15 were male and 2 were female (Table 1). The mean age of the patients in group I was 26.1 years. The age distribution was as follows: 2 patients under 19 years old, 12 patients between 20 and 39 years, and 2 patients between 40 and 59 years old. The mean age of the patients in group II was 36.1 years. There were 2 patients who were under 19 years old, 7 patients between 20 and 39 years, and 8 patients between 40 and 59 years old (Table 2).

**Table 1:** Gender distribution

Gender	Group I (*n* = 16)	Group II (*n* = 17)
Male	15 (94%)	15 (88%)
Female	1 (6%)	2 (12%)

**Table 2:** Age distribution

Age	Group I (*n* = 16)	Group II (*n* = 17)
1–19	2 (12.5%)	2 (12%)
20–39	12 (75%)	7 (41%)
40–59	2 (12.5%)	8 (47%)

In group I, the most common cause of fracture was slip down, followed by blown, collide, and traffic accident. In group II, slip down was most common as in group I, followed by collide, traffic accident, and blow order (Table 3). In group I, the most common fracture site was angle, followed by symphysis and subcondyle. Unlike in group II, the most common fracture site was symphysis, followed by angle, body, and subcondyle (Table 4). After the application of the plate, the meantime to removal was 335 days for group I, and the shortest period was 168 days while the longest period was 1000 days. Group II had an average of 349 days. The shortest period was 154 days, and the longest period was 987 days. See the table for the distribution of the patient’s plate application period for group I and group II (Table 5). Clinical data after plate removal of group I and group II showed no plate fracture, screw loosening, TMJ disorder, malunion, or infection in both groups (Table 6). Clinical examination of the patients showed no cases of malocclusion, occlusal discomfort, or sensory abnormality in both groups.

**Table 3:** Cause of injury

Cause of injury	Group I (*n* = 16)	Group II (*n* = 17)
Slip down	5 (31.3%)	10 (60%)
Traffic accident	3 (18.7%)	2 (12%)
Blow	4 (25%)	1 (6%)
Collide	4 (25%)	3 (16%)
Post-op	0 (0%)	1 (6%)

**Table 4:** Fracture site

Fracture site	Group I (*n* = 16)	Group II (*n* = 17)
Angle	12 (66.6%)	8 (40%)
Symphysis	4 (22.2%)	9 (45%)
Subcondyle	2 (11.1%)	1 (5%)
Body	0 (0%)	2 (10%)

**Table 5:** Plate duration period

Time(month)	Group I (*n* = 16)	Group II (*n* = 17)
< 6 months	2 (12.5%)	2 (12%)
6–12 months	10 (62.5%)	10 (59%)
> 12 months	4 (25%)	5 (29%)

**Table 6:** Clinical complications

	Group I (*n* = 16)	Group II (*n* = 17)
Plate fracture	None	None
Screw loosening	None	None
TMJ disorder	None	None
Malunion	None	None
Infection	None	None
Malocclusion	None	None
Numbness	None	None

## Discussion

We have reported the clinical application of YK plates and their short-term stability [9]. This study aimed to evaluate the long-term stability of YK plates through a 3-year follow-up. We compared patients who underwent plate removal with a YK plate and those who received plate removal with a conventional plate. The mechanical problems of the plate and the clinical problems of the patients were compared, and consequently, no significant problems occurred in both patients using the YK plate and patients using a conventional plate. To apply the plate in the control group, the plate was first placed on the desired position, and then the screw was applied. In the case of a fracture of the mandibular symphysis area, the view is good and the bone is located perpendicular to the gravity. Therefore, when the plate is positioned at the supine position, it maintains its position without additional fixation. Consequently, it is easy to apply a screw. However, in the condylar area and the mandibular angle fracture, the visual field is not good and the bone cannot be operated with the perpendicular to gravity. Therefore, when placing the plate on the surface of the bone, an additional fixture should be obtained by using a plate holder or the like, and then a screw should be applied. The volume of the plate holder itself makes the surgical field of view narrower. In addition, even when the plate is fixed, it often happens that the plate is slipped from the position to be fixed to another site, thereby increasing the operation time. To solve this problem, we have altered the shape of the hole in the plate to the shape of the keyhole through which the screw head can pass. This allows the screw to be applied first before applying the plate. After passing through the keyhole part of the plate with the applied screw, it is slid and then fixed. As a result, the plate is less likely to slip or fall, and the unnecessary operation time can be reduced.

Further, an additional fixing instruments such as a plate holder is not necessary, which helps to secure a visual field. For this reason, smaller incisions are made to ensure the field of view, and pulling of the retractor is reduced. This may lead to a reduction in trauma during the operation, which may help to speed up the recovery. When new medical devices and medical technologies are introduced, there is often a need for additional training period. However, in the case of YK plate, it is possible to substitute the conventional plate easily because it can be intuitively applied without specialized training if a surgeon has experienced operation using a traditional plate.

The majority of the patients were male in both groups I and II. This result is consistent to the report by Hanba et al. [13]. It is estimated that male is more active than female, so the probability of trauma is high [14, 15]. This can be deduced from the data on the reasons for the injury. In groups I and II, the cause of the injured woman was only slip down. All other traffic accidents, blow and collide, were male. No mechanical problems such as plate fracture and screw loosening occurred in all patients.

We reported the mechanical stability of the YK plate through finite element analysis using the orthognathic surgery model, and the study showed no significant difference between conventional miniplate and stress distribution [16]. This study, which compares conventional plate and YK plate at the time of plate removal, confirmed that the YK plate has a long-term clinical function.

## Conclusions

Developed for clinical convenience, the YK plate system with the screw inserted first and the plate applied did not cause any particular problems even for a long time from the mandible to the point of removal of the plate compared to the conventional plate.

## Data Availability

Please contact the author for data requests.

## Abbreviations

AO: Association for Osteosynthesis
ASIF: Association for the Study of Internal Fixation
YK: Yang’s Keyhole
